# G Protein-Coupled Receptors As Regulators of Localized Translation: The Forgotten Pathway?

**DOI:** 10.3389/fendo.2018.00017

**Published:** 2018-02-02

**Authors:** Aurélie Tréfier, Lucie P. Pellissier, Astrid Musnier, Eric Reiter, Florian Guillou, Pascale Crépieux

**Affiliations:** ^1^Biologie et Bioinformatique des Systèmes de Signalisation, INRA, UMR85, Physiologie de la Reproduction et des Comportements, Nouzilly, France; ^2^Déficit de Récompense, GPCR et sociabilité, INRA, UMR85, Physiologie de la Reproduction et des Comportements, Nouzilly, France; ^3^Plasticité Génomique et Expression Phénotypique, INRA, UMR85, Physiologie de la Reproduction et des Comportements, Nouzilly, France; ^4^CNRS, UMR7247, Nouzilly, France; ^5^Université François Rabelais, Tours, France; ^6^IFCE, Nouzilly, France

**Keywords:** G protein-coupled receptor, translatome, local translation, signaling, differentiation

## Abstract

G protein-coupled receptors (GPCRs) exert their physiological function by transducing a complex signaling network that coordinates gene expression and dictates the phenotype of highly differentiated cells. Much is known about the gene networks they transcriptionally regulate upon ligand exposure in a process that takes hours before a new protein is synthesized. However, far less is known about GPCR impact on the translational machinery and subsequent mRNA translation, although this gene regulation level alters the cell phenotype in a strikingly different timescale. In fact, mRNA translation is an early response kinetically connected to signaling events, hence it leads to the synthesis of a new protein within minutes following receptor activation. By these means, mRNA translation is responsive to subtle variations of the extracellular environment. In addition, when restricted to cell subcellular compartments, local mRNA translation contributes to cell micro-specialization, as observed in synaptic plasticity or in cell migration. The mechanisms that control where in the cell an mRNA is translated are starting to be deciphered. But how an extracellular signal triggers such local translation still deserves extensive investigations. With the advent of high-throughput data acquisition, it now becomes possible to review the current knowledge on the translatome that some GPCRs regulate, and how this information can be used to explore GPCR-controlled local translation of mRNAs.

## Introduction

When an endogenous ligand binds to its membrane receptor, it takes less than seconds to engage a dedicated signaling network, including second messenger production. Intertwined cascades of protein post-translational modifications are then activated and ultimately modulate gene expression. The latter relies on transcription of immediate early genes (IEGs) within 1 hour, and of genes requiring new protein synthesis and chromatin remodeling, which takes several hours. Subsequently, the corresponding transcription products need to be processed, exported through the nuclear pores, and transported in the cytosol to reach their ultimate destination, in specific cell compartments.

So far, numerous studies failed in finding a strict correlation between mRNA transcription, quantified by studying the transcriptome, and the cell protein content (1–5). Among other parameters (5, 6), gene expression also relies on protein neosynthesis from preexisting pools of mRNA, as initially observed in the early embryo (7). Importantly, mRNA translation takes place within minutes following extracellular signal action, since it is directly branched to the cell signaling network, which explains in part why it is temporally uncoupled from transcription in some instances. Hence, regulations at the level of translation lead to early modification of the cell protein content in response to subtle variations of their environment, mediated by growth factors, stress, or hormones (8).

Many extracellular signals exert their function by binding to G protein-coupled receptor (GPCR), not only hormones but also chemokines, neurotransmitters, lipids, amino acids, ions, light, odorant molecules, etc. These receptors are endowed with a flexible three-dimensional structure dynamically oscillating between active and inactive conformations that are stabilized by ligand binding and coupling to transducing partners (9). The conformational modifications of the receptors are directly sensed by G proteins and by β-arrestins that transmit the signal within seconds or few minutes to a complex intertwined signaling network (10–12), altering long-term gene expression and leading to cell-specific responses. Within this network, both G proteins and β-arrestins transduce signaling to control translation, either by impacting on the translation initiation complex or by altering the upstream signaling cascades that regulate the assembly of the translational machinery (10). In this review, we summarize the state-of-the-art knowledge of the impact of GPCRs on the translatome and address their role in spatially localized translation, in specialized cell types.

## Why is it Interesting to Assess the Impact of GPCRs on mRNA Translation?

mRNA translation covers two main cell requirements, i.e., cell proliferation and homeostasis on one hand (mainly quantitative control), and the production of cell-specific markers of the differentiated state (quantitative and qualitative control) on the other (13). Although some GPCRs are endowed with mitogenic activity, GPCR-regulated mRNA translation mainly operates in highly specialized cells such as neurons, retinal rods, gonadal Sertoli cells, endothelial cells, immunocompetent cells, among others, where these receptors may regulate a trophic function. For example, the follicle-stimulating hormone receptor (FSHR) promotes neosynthesis of paracrine factors essential for spermatogenesis to proceed, in the nurturer Sertoli cells of the testis (14). Similarly, in order to accommodate to the workload, or in pathological cardiovascular conditions, the endothelin ET_1_R enhances cardiomyocyte hypertrophy *via* mRNA translation, in addition to its acute vasoactive activity (15). Other GPCRs exert a trophic role on their target cells like adenosine and purinergic P2Y receptors in endothelial cells during angiogenesis and vascular remodeling [reviewed in Ref. (16)], or the neurotransmitter muscarinic (17) or GABA_B_ (18) receptors during brain maturation.

Notwithstanding, little is known about the mRNA-selective translation induced by GPCR activation. In differentiated cells, GPCR agonist binding leads to the regulation of the translation of selective mRNAs with minor, yet significant impact on global neosynthesis. For example, activation of the FSHR by FSH binding leads to the selective translation of the *vegf* and *c-fos* mRNA within minutes, without significant parallel effect on transcription (19). So far, whereas the regulation of transcription induced by GPCR activation has been investigated for a long time, the regulation of cell-type specific genome-wide translatome by GPCRs has been surprisingly poorly investigated.

## The Scarse Translatomes of GPCRs

From the late 2000s, whereas the number of publications on GPCR transcriptomes in various cell types and tissues has kept flourishing, only the translatomes of ET_1_R, GnRHR, LHR, mGluR_1_/_5_, D1 and D2 receptors in mammalian cells have been published (Table 1). Most of them have been obtained by polysome profiling (20). In this approach, mRNAs are separated according to the number of ribosomes they associate with. Practically, free mRNAs, the 40S and 60S ribosomal subunits and monosome/polysome-bound mRNAs sediment along a sucrose gradient, upon ultracentrifugation. mRNAs purified from each fraction are then analyzed by DNA microarray, or, nowadays, by NGS. Importantly, it becomes now possible to analyze actively translated mRNA *in vivo* by using the TRAP method (20). This approach consists in genetically modifying mice in order to label one protein of the 60S ribosomal subunit with a tag, in a cell-specific manner with the Cre-lox system (20). Following tissue extraction and immunoprecipitation with an anti-tag antibody, ribosome-bound mRNA can be identified, in one cell type entangled within a complex tissue (21).

**Table 1:** Main conclusions drawn from the study of the translatome of the endothelin receptor, the GnRH receptor, LH receptor, metabotropic glutamate receptors 1 and 5, dopamine receptors 1 and 2.

G protein-coupled receptor	Cell/tissue model	Methodology	Question addressed	Main conclusions	Reference
ET1R	Primary neonatal rat ventricular cardiomyocytes	Polysome profiling (Affymetrix μ-array)	Connection between early signaling and developed hypertophy	The ET1R signal propagates through the transcriptional network to promote the long-term phenotype-67% of variations in mRNA content are reflected at the translation level	(22, 23)
GnRHR	LβT2 pituitary cells	Polysome profiling (Affymetrix μ-array)	Is pausing a generalized response to UPR or are gonadotropin mRNAs specifically prone to translational pausing?	Selective pausing of some GnRHR target genes-Subtle regulation of translation to monitor protein quality and quantity	(25)
LHR	Leydig cell-specific in RiboTag mouse	Polysome profiling (Affymetrix μ-array)	Identify the transcripts that LH and FSH regulate *in vivo*, in the adult testis	LH regulates mRNA translation in the adult testis	(26)
mGluR1/R5	Primary mouse cortical neurons	Polysome profiling (Illumina RNA-seq)	Which specific mRNA are translated in mGluR-LTD?	eIF2α is a major effector of mGluR-LTD-Silences general translation while inducing mRNA-selective translation	(28)
DRD1	Mouse striatonigral neurons	Affinity purification of tagged ribosomes (Affymetrix μ-array)	Distinguish striatonigral from striatopallidal neurons on the basis of their translational profile	Identification of striatonigral-specific translated mRNA	(30)
DRD2	Mouse striatopallidal neurons	Affinity purification of tagged ribosomes (Affymetrix μ-array)	Distinguish striatonigral from striatopallidal neurons on the basis of their translational profile	Identification of striatopallidal-specific translated mRNA	(30)

### ET_1_R Translatome

The first GPCR translatome published was the one of the ET_1_R induced by endothelin-1 (ET1) in cardiomyocytes (22). Cardiomyocyte hypertrophy is associated with increased cell size and myofibrillar content and the authors investigated the ET_1_-dependent relationships between the early signaling/gene expression (IEG) and the late gene expression in the established phenotype. To evaluate how the variations in early transcriptional response to ET1 were reflected by mRNA translation, the profiles of total and polysomal RNAs have been compared. It appeared that 67% of variations in mRNA content were also reflected at the translational level. Hence, most of the mRNA transcribed from IEG appeared also translated in response to ET1. However, 17% of mRNA were increased to a greater extent in the polysome-associated pool than in the total transcriptome whereas some others were excluded, which is indicative of regulations of the translational machinery by ET1R-induced signaling to promote mRNA-selective translation, as the authors confirmed later (23).

### GnRHR Translatome

More recently, the unfolded protein response (UPR), a mechanism that maintains protein quality in secretory cells, has been explored in the LβT2 gonadotrope cell line derived from the anterior pituitary. This study has revealed that GnRHR activation enhances the translation of selective mRNA, such as the one encoding Dusp1, which is assumed to participate in the decoding of GnRH pulsatility in gonadotrope cells (24). Simultaneously, ligand-bound GnRHR also appeared to induce a pause in the translation of several mRNA involved in reproduction, such as the ones encoding the LH β and α chains of gonadotropins, in the endoplasmic reticulum-associated polysomes (25). By these combined means, GnRHR stimulation would fine-tune both protein quality and quantity in secretory gonadotrope cells.

### LHR Translatome

By using the TRAP technology, the effect of FSH and LH on the regulation of spermatogenesis by somatic gonadal cells has been assessed (26). AMH-cre and Cyp17i-cre mice have been crossed with mice expressing HA-tagged RpL22, in Sertoli cells and in Leydig cells, respectively. Polysome-associated mRNA were identified on Affymetrix microarrays in both cell types in isolation. While no increase in the FSH translatome was observed, LH altered the basal Leydig cell translatome. For example, as soon as after 1 h, the LH signal enhanced the translation of the Nr4a1 et Egr1 transcription factors, and of the Rgs2 cell cycle regulator. After 4 h, the number of translated mRNA still increased, showing that, in the adult, LH regulates mRNA translation in the seminiferous tubules.

### mGluR_1_/_5_ Translatome

The translational control by GPCRs has been the most frequently studied in neurons. One of the reasons for this interest is that the subcellular localization of translation in these highly polarized and organized cells is a critical aspect of GPCR physiological function in the nervous system. Hence it deserves consideration, particularly in synaptic plasticity (see below), such as long-term depression (LTD). LTD is an activity-dependent decrease in synaptic tone that is mediated notably by ionotropic or metabotropic glutamate receptors (iGluR and mGluR, respectively). mGluRs, but not iGluRs, are GPCRs, and at the hippocampal synapses, the specific activation of group I mGluRs (mGluR_1_ and mGluR_5_) induces LTD *via* local protein neosynthesis (27). Furthermore, the phosphorylation of the eIF2α translation initiation factor is a major effector of mGluR-induced LTD in these neurons (28). By combining polysome profiling and deep-sequencing, the authors showed that 3,5-dihydroxyphenylglycine (DHPG), the selective agonist for mGluR_1_ and mGluR_5_, induced eIF2α phosphorylation that correlated with not only a general silencing of translation but also significantly enhanced the selective translation of several mRNA such as the one encoding oligonephrin 1 (Ophn1). Injection of Ophn1 shRNA in mouse hippocampus prevents LTD formation and impairs mouse performance in a hippocampal learning task. Altogether, mGluR_1_/_5_ activation, eIF2α phosphorylation, and Ophn1 translation contribute to remove AMPA-type Glutamate receptors (AMPARs) from the cell surface, leading to depression of AMPAR-mediated excitatory postsynaptic current (28). mGluR-LTD-dependent local translation of activity-regulated cytoskeleton-associated protein (Arc) in the dendrites is also involved in AMPAR endocytosis (29).

### Approaching the D_1_ and D_2_ Receptor Translatome by TRAP Assay

Mouse lines have been genetically engineered to express a GFP-tagged ribosomal protein L10a under the control of a defined locus, in a specific cell type, by using BAC vectors (30). Following anti-GFP immunoprecipitation, ribosome-bound transcripts have been sequenced. This TRAP approach has been initially designed to discriminate the translatome of different neuronal subtypes that are morphologically indistinguishable, namely striatonigral and striatopallidal medium spiny neurons using 2 GPCR loci: cell-type selectivity of L10a-GFP expression is controlled either by dopamine D_1_ (striatonigral) or D_2_ (striatopallidal) receptor locus (30). By these means, specific mRNA associated with ribosomes of either striatopallidal (e.g., Adk, Plxdc1, BC004044, et Hist1h2bc) or in striatonigral (e.g., Slc35d3, Zfp521, Ebf1, Stmn2, Gnb4, et Nrxn1) neurons have been identified. Hence, the TRAP method has paved the way for *in vivo* studies of mRNA translation profiling in selective cell-types of native tissues, in physiological or pathological conditions.

The discovery of GPCR translatome in increasing number should help to identifying new secondary structures in mRNA UTRs. By these means, new advances should be made on how some mRNA subpopulations bearing similar binding motifs for common cargos may be transported in the intracellular space, to be cotranslated locally.

## Involvement of GPCR in Local Translation

Translation of selective mRNAs in specific subcellular location is an important contributor to cell regulatory processes, associated with morphological asymmetry. This permits the local production of multiple units of the same protein from a single mRNA molecule, hence limiting energy consumption, as initially demonstrated at the genome-wide level during Drosophila early embryogenesis (31). In addition, local translation enables rapid protein synthesis during neuronal plasticity and is a convenient means to discriminate the activated synapse(s), at long distance from the cell body. Similarly, in migrating cells, directional motility toward a chemoattractant gradient is mediated by spontaneous cell polarization where proteins are differentially translated in the protrusions *versus* the cell body (32, 33).

To date, little is known on how extracellular signals control local translation. In the nervous system, both neurotransmitters and growth factors are involved in synaptic plasticity and axonal growth, by binding to GPCR or to growth factor receptors such as neurotrophin receptors (34) or BDNF receptor (35, 36). These classes of receptors share common signaling pathways (mTOR, ERK1/2) potentially involved in local mRNAs translation, but their direct coupling to different adapter proteins (respectively, β-arrestins and G proteins vs. SHC-1 or IRS-1) might affect the dynamic properties of the underlying signaling network. Despite these fragmentary data (Table 2), so far, how an extracellular GPCR ligand dictates where in the cell and when a mRNA is to be translated into a physiologically relevant protein remains, for the main part, an unresolved question.

**Table 2:** Summary of the G protein-coupled receptors that regulate local translation processes, in different biological settings.

Receptor	Stimulus	Main process	Biological model	Pathway	Reference
mGluR5	DHPG	Synaptogenesis	E16.5 hippocampal neurons	TLS/FUS	(54)
DRD1/DRD5	Dopamine	Synaptic transmission	Hippocampal neurons	cAMP/PKA	(56)
ADRB1	Isoproterenol	LTP	CA1 hippocampal neurons	PKA/ERK	(57)
mGluR1	Glutamate	Axon migration	Axons of developing brain	Ca^2+^/mTOR	(66)
5HTR	Serotonin	Long-term facilitation	Axons of Aplysia sensory neurons	eEF1A	(67)
mGluRs	Glutamate	Myelination of electrically active axons	Oligodendrocytes	Fyn	(69)
CXCR4	SDF1	Cell migration	Fibroblasts	eIF2B	(71)
mGluR1,5	DHPG	LTD	Dendrites of CA1 pyramidal neurons	ERK/PI3K/Mnk1/eIF4E/4E-BP/eIF2α	(28, 89)
mGluR1	PP-LFS	Synaptic plasticity	Mossy fibers of CA3 pyramidal neurons	βarr2/Src/pERK	(85)
mGluR5	PP-LFS	Synaptic plasticity	CA1 pyramidal neurons	βarr2	(85)
mGluR5	CDPPB	Neuronal plasticity (LTD)	Hippocampal slices	βarr2/pERK/FMRP	(101)
ADRB1	Object recognition memory reactivation	Memory reconsolidation	Entorhinal cortex	βarr2/pERK	(102)

### General Principles

**Figure 1:**
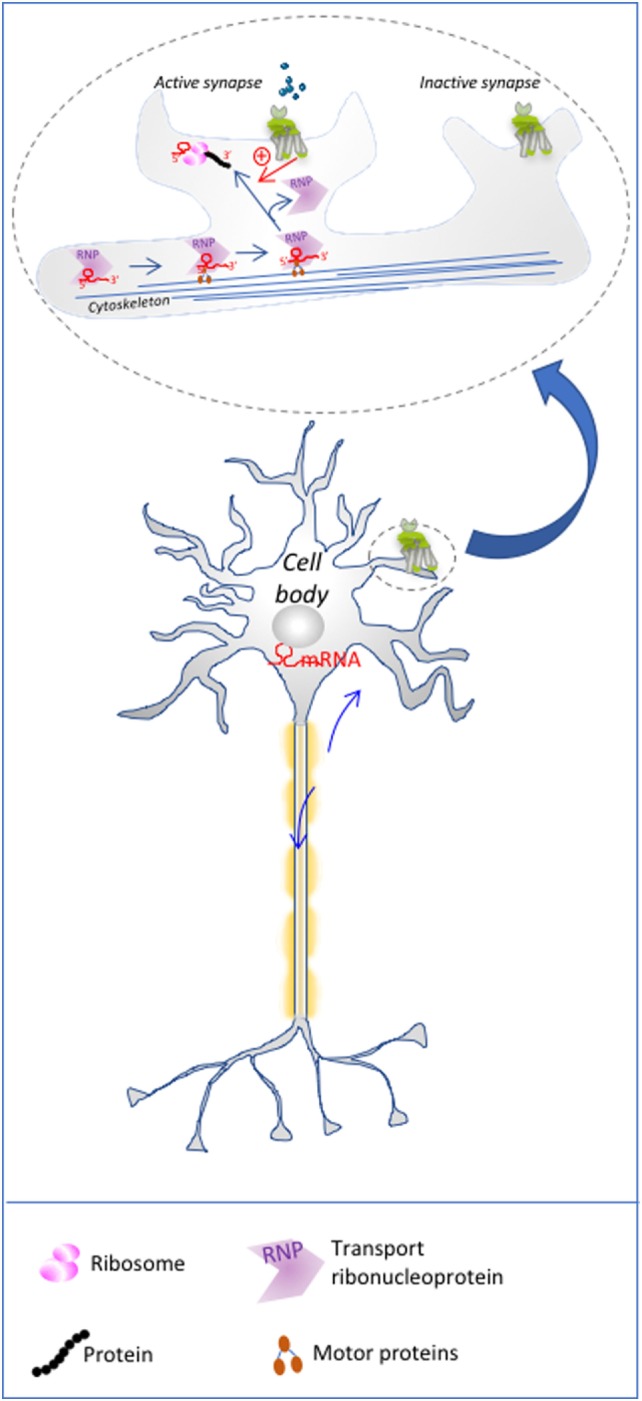
Local translation in neurons: mRNA to be translated are transported to dendritic spines, where mGluR postsynaptic activation relives the inhibitory action of ribonucleoprotein granules (RNPs) such as fragile X mental retardation protein on mRNA, *via* the activation of signaling pathways. This process restricts local translation only to the active spines of a single neuron. The upper part of the illustration is a magnification of schematized dendritic spines.

For site-specific translation to occur, mRNAs need to be transported from the nucleus to the specific intracellular location where they will be ultimately translated, while preventing premature translation during their transport. In the nucleus, a ribonucleoprotein granule (RNP) gathers ribonucleoproteins (RBPs) and mature mRNAs (Figure 1). RBPs may recognize a structural motif, a so-called zipcode, in the 3′ or 5′ UTRs of the mRNAs, through a canonical RNA-binding domain (37). Recognition of mRNA sequences by proteins with low-complexity domains has emerged as a means to dynamically integrate mRNA into RNP granules to form an mRNP (38, 39). Cross-linking immunoprecipitation (CLIP) analyses have revealed that some RBPs, such as fragile X mental retardation protein (FMRP), are able to bind hundreds of different mRNAs, while others only bind one. mRNA passage through the nucleopores is facilitated by export receptors such as the TAP-15 complex that associate directly with mRNA-bound RBP such as Aly/REF adaptors, and dissociate from the RNP granule once in the cytoplasm (40). Then, the silent mRNP complex is transported by motor proteins along the actin or microtubule cytoskeleton tracks toward the site of translation. Among others, the Staufen protein could be a general regulator of mRNA transport (41), in *Drosophila* embryo as well as in neurons (42). Another well-described RBP is the ZBP1 protein that silences the β-actin mRNA to avoid its premature translation in the cytoplasm, prior to its arrival at fibroblast protrusions (43). Upon arrival at the site of translation, the Src protein phosphorylates ZBP1, which leads to its dissociation from the mRNP, hence making the mRNAs accessible to the translational machinery (44). Extracellular signaling events are presumably the key step that unmasks the mRNAs enabling their local translation. Besides budding yeast, *Xenopus* oocyte, and *Drosophila* embryo, cells of the nervous systems have become the paradigm to study localized mRNA translation in Mammals. Notably, the mRNA content is qualitatively different not only in dendrites, axons, and cell body but also among several dendritic spines of the same dendrite. Likewise, the distribution of neuronal GPCR content differs in the plasma membrane of the soma, the dendrites or the axon terminals (45). For example, in the *nucleus accumbens*, D_1_ and D_5_ dopaminergic receptors are located mainly in dendritic spines and axonal terminals, respectively (46). Their distribution determines their postsynaptic function, their responsiveness to local neurotransmitter release and, more generally, their role in regulating neuronal activity (Figure 1). In synapses, silencing by components of the miRNA processing machinery have also been invoked in translational repression. Interestingly, neuronal activity could relieve this silencing by degrading miRNA locally, as shown in *Drosophila* (47).

### Local Translation in Neurites

The hypothesis that mRNA might be transported to specific subcellular compartments in neurons has emerged from the observation that the mRNA content of dendrites and axons differs. mRNA prelocalization would enable rapid production of proteins at the activated synapse, independently of the inactive synapses of the same neuron, which is important for synaptic plasticity and memory. For example, the mRNA encoding microtubule-associated protein 2 (MAP2) is transported to the dendrites, whereas the tubulin mRNA is located in the axons and dendrites (48). Accordingly, the presence of polyribosomes, translation factors and mRNA at the base of dendritic spines has been reported (49–51). In addition, protein synthesis inhibitors have revealed the central role of local translation in synaptic plasticity, in the dendrites of CA1 pyramidal neurons (52). The mRNA content of the dendrites is dynamically regulated. mGluRs are the GPCRs the most extensively reported to control local translation in neurons. Their stimulation by the DHPG mGluR_1_/_5_ agonist leads to actin-related protein mRNA redistribution to dendritic spines, that permits spine remodeling upon synaptogenesis. This process is in part mediated by the RNA-binding protein TLS/FUS (53, 54). TLS/FUS harbors a low complexity sequence domain involved in the dynamic aggregation of RNA-binding proteins to form RNP granules (38, 55). These regulatory events lead to the rapid withdrawal of excitatory synapses in the neurons of the hippocampus and striatum. Furthermore, in the hippocampus, dopamine D_1_/D_5_ receptor signaling alters synaptic plasticity *via* the translation of the GluR1 subunit of AMPA iGluR, which leads to local activity at synapses that were otherwise silent (56). Likewise, mRNA translation of AMPAR is stimulated during β1-adrenergic receptor-primed long-term potentiation in the CA1 hippocampal region, in a PKA and ERK-dependent manner (57), ultimately leading to its enhanced location at the plasma membrane and synaptic incorporation.

Protein synthesis could serve as one of the gates for synaptic plasticity that physiologically operates when mGluRs are activated. *FMR1* knock-out mice models are useful tools to investigate activity-dependent translation in synaptic plasticity. In the fragile X syndrome, a monogenic form of autism spectrum disorder, the loss-of-function of FMRP encoded by the *FMR1* gene leads to excessive translation of proteins, that would otherwise be rate-limiting for synaptic remodeling. Examination of the phenotype reveals defects in synaptic plasticity associated with an exacerbated mGluR-LTD (58–60). These observations are consistent with a direct silencing role of FMRP (Fragile X mental retardation protein) on neuronal translation (61). For example, the *Chrm4* mRNA encoding the muscarinic acetylcholine receptor 4 (M_4_) is excessively translated in *Fmr1^−/y^* mice occluding mGluR_1_/_5_ activation (62). Unexpectedly, enhancement of M_4_ activation is required to correct the excessive translation and mGluR-LTD pathological phenotype of these mice.

FMRP is an RNA-binding protein involved in mRNA trafficking and translation. The *FMR1* knock-out mice model has also highlighted the association of aberrant synthesis of proteins involved in mGluR-dependent synaptogenesis such as PSD-95, MAP1b or CAMKIIα and defective long-term plasticity (63–65).

In contrast to local translation in dendrites, much less is known about local translation in axons. Both dendritic spines and axonal terminals are specialized subcellular compartments located far away from the cell body. In growing axons, local mRNA translation might be involved in collapse or expansion of the growth cone during axon guidance whereas in mature axons, it might support regeneration of the nervous fiber upon injury. Most of these responses are dynamically regulated by signals emanating from non-GPCR receptors. However, in cortical neurons of the developing brain, it has been reported that glutamate enhances local protein synthesis, by interacting with both iGluRs and mGluRs in axons (66). In addition, in an *in vitro* model of *Aplysia* sensory/motor neurons, the local translation of the *eEF1A* mRNA has been involved in maintaining newly grown synapses, a prerequisite for long-term facilitation required for memory storage. Serotonin stimulation supports this site-specific targeting of the eEF1α mRNA if it is applied at the synaptic site solely (67).

### Local Translation in Glial Cells

Cells of the nervous systems are highly interconnected and local translation can be viewed as one means whereby they communicate. This is the case at synaptic junctions, including synaptic communications between neurons and oligodendrocytes. These glial cells synthesize the myelin sheath, to wrap the axons, in order to increase the propagation speed of the nervous influx. The mRNA that encodes myelin basic protein (MBP), a major component of myelin, is transported to distal regions of oligodendrocytes for local translation and delivery to the membrane of the adjacent axon (68). Vesicular release of glutamate from activated axons of mouse dorsal root ganglion neurons has been shown to stimulate the local translation of MBP, by using a photoconvertible fluorescent protein. The glutamate signal is sensed by both AMPA iGluR and mGluRs on oligodendrocytes (69). Recently, local translation in distal perisynaptic processes of astrocytes has also been observed (70), suggesting that the proteins these cells secrete are produced locally prior to release. However, the sensitivity to neurotransmitters of this process has not been explored yet.

### Local Translation in Migrating Cells

More than 1,000 mRNAs have been shown to exhibit site-specific translation in migrating fibroblasts (33), which supports a broader role of this process in cellular monitoring of the proteome in time and space than initially appreciated. Beside promoting the migration of a wide spectrum of motile cells of hematopoietic type, the CXCR_4_ chemokine receptor also triggers the migration of non-hematopoietic cells, such as fibroblasts. Local translation in migrating fibroblasts is exemplified by the β*-actin* mRNA, which displays an asymmetric distribution at the leading edge where its mRNA is translated upon ZBP1 release, as described above, in order to enable cell motility. Recently, CXCR_4_ has been shown to interact with the eukaryotic initiation factor 2B (eIF2B) (71). This protein is an exchange factor that negatively regulates translational efficacy. Binding of the CXCR4 agonist, SDF1/CXCL12, leads to the dissociation of eIF2B from the receptor. This observation suggests that the local release of this translation factor could then be utilized locally, close to the plasma membrane, to enable local translation of the β*-actin* mRNA. Early work already showed that eIF2B also interacts with the β_2_-adrenergic receptors in protruding regions of the cell membrane (72).

Evidence is lacking that other GPCRs might be involved in local translation of actin-related proteins needed for cell motility. But, noteworthy, β-arrestins, major effectors that regulate the efficacy, duration and location of GPCR-responsive signaling pathways (73, 74), are involved in chemotaxis (75). Despite their ability to redistribute signaling components to selective subcellular compartments, a role of these adaptor proteins in local translation in migrating cells has not been addressed yet.

As discussed above, it appears clearly that most studies to decipher the control of site-specific mRNA translation by GPCR have been undertaken in neurons. However, local translation processes virtually take place in any cell type, such as epithelial cells from the kidney, the gut, the skin, provided that they exhibit some degree of polarization and functional specialization. For example, Sertoli cells represent an ideal model for studying local mRNA translation. They are polarized cells that constitute the seminiferous tubules of the male gonad and the intimate association they undergo with the successive steps of spermatogenesis has prompted investigators to refer to this histological architecture as the “testicular synapse”. One of their major regulatory GPCR is the FSHR that stimulates Sertoli cell anabolic activity to provide each spermatogenic cell with the adapted complement of paracrine factors. Recently, the FSHR has been reported to promote mRNA-selective translation of some mRNAs such as the *vegfa* mRNA (19) that is involved in spermatogonia renewal occurring at their basal region. However, despite many analogies to neurons, hormone-induced local translation has never been demonstrated in these cells.

## Signaling Involved in GPCR-Transduced Regulation of Localized Translation

In Sertoli cells, signaling pathways such as PKA and PI3K/mTOR have been shown to regulate proteins of the eIF4F initiation complex (76, 77). Interestingly, local regulation of Rap1 by the Mex3b RNA-binding protein is required for maintaining cell polarity (78). Regarding Sertoli cell/germ cell contacts, FSH-induced PTEN translation occurs at the apical pole of Sertoli cells during spermiation (79). Symmetrically, the kit-L is produced at the basal pole, a region in close contact with spermatogonia that express c-kit (80).

In polarized renal epithelial cells, agonist-induced adenylate cyclase activity is restricted to basolateral or apical regions where α_2B_- and α_1_-adrenergic receptors, respectively reside (81). Activation of GPCR-dependent p90RSK pathway in selective subcellular compartments may enable de-repression of mRNAs once arrived at their specific site of translation.

**Figure 2:**
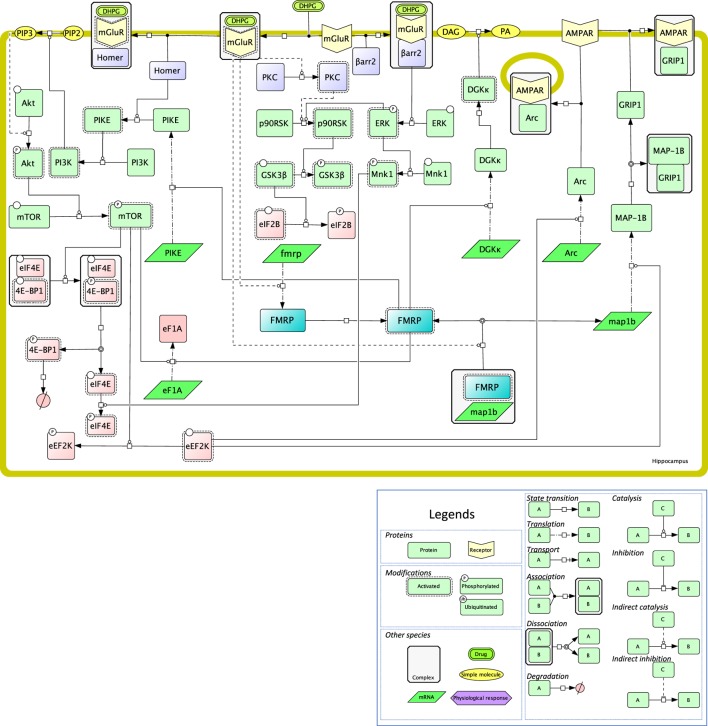
Mechanistic map of the signaling pathways involved in local translation mediated by class I mGluRs in neurons. The biochemical reactions were edited in the Cell Designer format, that ascribes to each reaction and molecular species a precise semantics, as developed in the legend. Signaling proteins are in light green, RNAs are in dark green, proteins of the translational machinery are in pink, receptors are in yellow, small molecules are in orange, fragile X mental retardation protein (FMRP) is highlighted in blue, transducing mechanisms are in purple (PKC is activated *via* Gq). Plain lines indicate direct reactions, while dashed lines indicate indirect ones. All the reactions shown on this figure have been published and are discussed in the text.

Again, the signaling effectors of local translation have been mostly investigated in the case of mGluR activation (82). The ERK MAP kinase pathway is critical in mediating mGluR-dependent protein synthesis in physiological as well as pathological conditions (83). mGluR stimulation of hippocampal synaptoneurosomes leads to the recruitment of one of ERK substrates, p90RSK, to the ribosomes (84). The phosphorylation of glycogen synthase 3β (GSK-3β) would relieve eIF2B-mediated translational inhibition (Figure 2). Another target of ERK that is also stimulated upon mGluR activation (DHPG binding) is Mnk, a kinase that phosphorylates the cap-binding protein eIF4E (Figure 2). In CA3 hippocampal neurons, this pathway has been shown to be mostly regulated by β-arrestin 2 to regulate synaptic plasticity (85).

In addition, DHPG-induced LTD in the dendrites of hippocampal neurons is also mediated by the PI3 kinase/mTOR pathway (86), presumably as a consequence of mGluR binding to the postsynaptic adaptor protein Homer (87) (Figure 2). PIKE has been proposed as a molecular link between Homer and PI3K activation in neuronal survival, and its role in mGluR-mediated local translation has been proposed (88). PI3K/mTOR activation during LTD leads to the inhibitory phosphorylation of 4E-BP, the inhibitor of eIF4E (89) (Figure 2). In agreement, 4E-BP2 knock-out mice exhibit exacerbated mGluR-LTD, hence confirming the negative regulatory role of this translation inhibitor on synaptic activity. Other well-known targets of the PI3K/mTOR pathway, such as ribosomal protein S6 (rpS6) (90) or eEF2K (91), are also engaged in protein translation upon mGluR activation in the hippocampus (Figure 2). In this brain region, DHPG enhances locally in dendrites the translation of EF1A (92), as well as phosphorylation (90), that is involved in the assembly of the translational preinitiation complex (93). In mGluR-LTD, rpS6 phosphorylation most likely results from the activation of RSK, and not of p70S6K, as it is not altered in S6K1-deficient mice (90).

More generally, type I mGluRs maintain synaptic plasticity by provoking the rapid elimination of excitatory synapses in the hippocampus and in the striatum. As indicated above, the FMRP protein plays a pivotal role in regulating these processes in dendrites, by modulating local protein translation in classical mGluR-LTD (94). For example, the *MAP1B* mRNA colocalizes with FMRP granules at hippocampal synapses neurons and its translation is stimulated by eEF2K in DHPG-stimulated neurons (91, 95) (Figure 2). MAP1B, a component of the cytoskeleton, is involved in the rapid internalization of AMPA iGluR during mGluR-LTD (96), by disrupting the interaction of the AMPA GluR2 subunit with GRIP1 (91). In parallel, mGluR activation during LTD also relieves the inhibition that FMRP exerts on the translation of the *Arc* mRNA at dendritic spines (97) (Figure 2). Together with GRIP dissociation from AMPA, Arc mediates a postsynaptic endocytic pathway that controls AMPA trafficking (98). Dynamic activation/deactivation of FMRP could in part result from the interplay between mTOR/PP2A (99) and CK2 (100), that temporally regulate its phosphorylation level. β-arrestin 2 appears to mediate the detrimental effect of FMRP on translation, by activating the ERK MAP kinase signaling module (101). β-arrestin 2-dependent translation is also involved in memory reactivation mediated by β1-adrenergic receptor, but whether mRNA translation is localized is not clear to date (102).

Fragile X mental retardation protein regulates translation negatively in most instances (61, 103) but also sometimes positively. More precisely, combined CLIP and TRAP assays have recently revealed that FMRP enhances the translation of the diacylglycerol kinase (DGKκ) mRNA that encodes a protein involved in mGluR signaling, among numerous other mRNAs involved in synaptic plasticity (104) (Figure 2). DGKκ, a member of the DGK enzyme family, is involved in spine maintenance most likely through the multiple effectors of DAG- and PA-mediated signaling (105).

A recent model has proposed that, beside targeting the rate-limiting step of translation initiation, synaptic mGluR signaling also acts on stalled polyribosomes, which optimizes the speed and efficacy of translation (106). This effect is mediated by UPF1, an RNA-helicase associated with the STAU2 RNA-binding protein (107).

As for axonal local translation, glutamate stimulates translation by binding to AMPA receptors and metabotropic glutamate receptors, thus activating Ca^2+^ and mTOR signaling (66). Besides, the interaction between glial cells and axons involves kinases of the Src family, such as Fyn, that could regulate *MBP* mRNA local translation to promote axons myelination (69, 108).

Beside the long-distance signaling to the translational machinery induced by membrane-bound receptors, an alternative/addition model has proposed that deleted in colorectal cancer (DCC), a receptor for netrin, forms a complex with components of the translational machinery at the membrane of neurons (109). This model will plausibly be generalized to other membrane receptors in the future, since ribosomes or translation initiation factors have been observed in their close vicinity, as illustrated above with CXCR4 and eIF2B, for example.

## Concluding Remarks

Many questions remain open on the signaling mechanisms that mediate GPCR-dependent local translation, because, by essence, their activity should also be localized. In support of this assumption, FMRP has been shown to control the localization of the mRNA encoding the p110 β catalytic subunit of PI3K at the synapse, where mGluR activation enhances p110 β local translation and PI3K activity (110).

Local translation appears as a key regulator of early gene expression in polarized cells, that is, in cells that engender differential responses in their different (sub)membrane region as a function of the connexions/relationships they have with their neighboring cells. This is typically the case of the neuronal network and of the Sertoli cells/germ cells assembly. Local translation responds to the signaling network generated by extracellular signals, among which some target GPCRs. Since GPCRs constitute a prominent class of therapeutic targets, the question is raised as to whether biased ligands may modulate the pool of local mRNAs. Biased ligands target various signaling components with variable efficacy within the total repertoire mediated by a given receptor when compared to the physiological ligand (111, 112). This process depends on the high level of conformational plasticity of GPCRs. Such tools are attractive candidates that could compensate dysregulated local translation at the site where the endogenous ligand is released to restore synaptic plasticity, in pathologic conditions, like the Fragile X syndrome, for example by dampening mGluR signaling (110). In the gonad, there are potential applications in the control of puberty onset or in the preservation of the male fertility by sustaining the renewal of the spermatogonial pool.

## Author Contributions

AT and PC wrote the article with significant contributions of the other authors. PC supervised the work.

## Conflict of Interest Statement

The authors declare that the research was conducted in the absence of any commercial or financial relationships that could be construed as a potential conflict of interest.
